# Multitarget Neuropharmacological Actions of *Drimycarpus racemosus* Hook. f. GC–MS‐Guided In Vivo and In Silico Evidence of Anxiolytic and Antidepressant Potential

**DOI:** 10.1155/tswj/7306249

**Published:** 2026-08-13

**Authors:** Md. Rakibul Islam, Md. Sajib Ali, Priota Islam Meem, M. A. Rafi, Sanjana Haque Esha, Md. Mehedi Hasan, Payar Hossain, Abdullah Ripon, Nilufar Sultana

**Affiliations:** ^1^ Department of Pharmacy, Manarat International University, Dhaka, Bangladesh, manarat.ac.bd; ^2^ Department of Pharmacy, Faculty of Biological Science, University of Chittagong, Chattogram, Bangladesh, cu.ac.bd

**Keywords:** 4UUJ, antidepressant, anxiolytic, *Drimycarpus racemosus*, molecular docking

## Abstract

Anxiety and depression are significant neurological disorders with limited treatment efficacy and notable side effects associated with conventional synthetic drugs. This study investigated the anxiolytic and antidepressant potential of the methanolic leaf extract of *Drimycarpus racemosus* Hook. f. (MEDR) using both in vivo behavioral models and in silico molecular docking approaches. GC–MS analysis of MEDR identified 39 phytochemical constituents, including bioactive terpenoids, phenolic compounds, and fatty acid esters. In vivo experiments were conducted (*n* = 5 per group) on Swiss albino mice randomly divided into control, standard (diazepam 1 mg/kg), and treatment groups receiving MEDR (200 and 400 mg/kg) using the elevated plus maze (EPM), hole‐board test (HBT), light–dark box (LDB), and forced swim test (FST). MEDR exhibited significant dose‐dependent effects. At 400 mg/kg, MEDR significantly increased time spent in the open arms of the EPM by approximately 89% (140.0 ± 1.79 s vs. 74.2 ± 2.71 s in control, ∗*p* < 0.05) and number of open‐arm entries (17.2 ± 1.43 vs. 10.4 ± 1.57) while markedly reducing immobility time in the FST by ~44% (102.2 ± 1.16 s vs. 182.6 ± 1.94 s in control, ∗*p* < 0.05). These effects were comparable to the standard drug diazepam (1 mg/kg). Molecular docking studies against the potassium channel (PDB ID: 4UUJ), GABAA receptor (PDB ID: 6X3X), and human serotonin transporter (hSERT, PDB ID: 5I6X) revealed that *β*‐sitosterol acetate and 2‐[4‐cyclohexylbutanoylamino]‐3‐chloro‐1,4‐naphthoquinone exhibited higher binding affinities than diazepam across all three targets. The combined in vivo and in silico findings indicate that *D. racemosus* exerts multitarget neuropharmacological effects, primarily through modulation of GABAergic and serotonergic systems. These results highlight the phytochemical richness and therapeutic promise of MEDR as a potential source for developing safe, plant‐based neurotherapeutics for anxiety and depression.

## 1. Introduction

Anxiety and depression are primarily classified as psychiatric disorders, although they involve complex neurobiological mechanisms and may occur as comorbid conditions in various neurological diseases. Mental health issues have emerged as a major public health concern in Bangladesh, as in many other countries. Conditions such as depression, anxiety, stress, and sleep disorders are increasingly recognized among the population [1]. Epidemiological studies indicate that over 20% of the global population is impacted by these conditions [2]. For example, approximately 40% of FDA‐approved medicinal chemicals, such as vinca alkaloids and taxol, are of natural origin [3]. Stress and oxidative imbalance are significant contributing factors. Depression raises the risk of physical illness, morbidity, and mortality, whereas anxiety can become a chronic, clinically significant condition. When occurring together, anxiety and depression often exacerbate prognosis, reduce responsiveness to pharmacological treatment, and elevate the risk of suicidal behavior.

Although the precise etiologies of anxiety and depression remain unresolved, multiple factors have been implicated, including genetic, environmental, biological, and psychological influences [4]. A key contributor to redox imbalance (oxidative stress) is the deterioration of the body′s antioxidant defense systems. Redox imbalance is implicated in the pathogenesis of numerous disorders. Oxidative stress has been identified as a pivotal element, wherein the overproduction of reactive oxygen species (ROS) interferes with neuronal signaling and results in cognitive impairment. In this context, combining antipsychotic medications with antioxidant supplements may help reduce ROS‐related CNS injury and could be a promising treatment approach [5].

Conventional treatment strategies rely largely on antidepressants and anxiolytics. The use of benzodiazepines as a primary treatment for anxiety has decreased due to concerns regarding dependence and tolerance. Antidepressants, such as selective serotonin reuptake inhibitors (SSRIs) and serotonin–norepinephrine reuptake inhibitors (SNRIs), serve as the primary treatment for depression and anxiety [6]. These drugs are often constrained by a delayed onset of action, variable efficacy, and adverse effects, including insomnia, gastrointestinal disturbances, neurological complications, and an elevated risk of seizures. This has stimulated an increasing investigation into safer and more effective alternatives that possess both anxiolytic and antidepressant properties.

Medicinal plants have been used for healing since ancient times, long before synthetic drugs were discovered. They are still important in primary healthcare because they are safe, affordable, and effective [7]. Research indicates that numerous plants possess bioactive compounds with potent pharmacological effects, aiding in the management of chronic diseases. Certain renowned herbs, including *B. monnieri*, *L. angustifolia*, and *C. sativus*, exhibit notable anxiolytic and antidepressant effects comparable to contemporary pharmaceuticals, highlighting the potential of phytochemicals in the treatment of neuropsychiatric disorders [8]. Because of their pleiotropic pharmacological characteristics and very minimal side effects, natural products have drawn a lot of interest as possible multitarget therapeutic agents. Through the simultaneous regulation of many signaling pathways and neurotransmitter systems, a variety of bioactive chemicals produced from plants have shown neuroprotective, antioxidant, anti‐inflammatory, anxiolytic, and depressive properties [3, 9]. The therapeutic potential of phytochemicals in treating complex neuropsychiatric illnesses, when the efficacy of single‐target medications is limited by multifactorial pathophysiology, has been shown by recent investigations. As a result, medicinal plants with a variety of bioactive components are good options for creating safer and more potent neurotherapeutic drugs [10]. Current research focuses on plant‐derived bioactive compounds to expand pharmacological applications, especially when traditional discovery is slow or challenging. Consequently, in silico methods are increasingly used to characterize molecules chemically and structurally and reduce candidate failures.

The *Drimycarpus racemosus* Hook. f. is a species of evergreen tree that is a member of the Anacardiaceae family. It is native to the countries of Nepal, Vietnam, Myanmar, India, Thailand, and Bangladesh, as well as several areas in southern China [11]. Locally, it is referred to by names such as kodi‐barela, nala‐amshi, and khali [12]. This species is commonly utilized in traditional medicine for treating skin diseases. Although it has a history of use in ethnomedicine and is ecologically widespread, there is a striking lack of scientific evidence supporting its pharmacological potential. The majority of current research is focused on the morphology and taxonomy of plants. There have been no reports of review, phytochemical profiling, or bioactivity assessment for this species yet. The lack of scientific validation for its bioactive components and therapeutic potential reflects a significant research gap. Moreover, although other members of the Anacardiaceae family exhibit significant antioxidant, anti‐inflammatory, and neuroprotective properties, *D. racemosus* has been predominantly neglected in terms of its pharmacological characteristics. Consequently, the objective of the current study was to conduct a comprehensive examination of the neuropharmacological activities and phytochemical constituents of the methanolic leaf extract of *D. racemosus,* with a particular emphasis on its anxiolytic and antidepressant potential, using a combination of in vivo and in silico methods.

This study is aimed at analyzing the antidepressant and anxiolytic effects of the methanol extract of *Drimycarpus racemosus* (MEDR) leaves using in vivo models and in silico analysis. We focus on target molecules, specifically the potassium channel (PDB ID: 4UUJ), the GABAA receptor, and the human serotonin transporter (hSERT) to identify the bioactive components that have therapeutic activities. The results would then provide a scientific basis for the traditional use of the plant and facilitate the identification of new therapeutic candidates for the treatment of anxiety and depression.

## 2. Materials and Methods

### 2.1. Chemicals and Reagents

Sigma–Aldrich (St Louis, Missouri, United States) supplied the following chemicals: methanol, dimethyl sulfoxide (DMSO), and distilled water. Furthermore, Square Pharmaceuticals Ltd. in Bangladesh supplied diazepam. This study employed solely analytical‐grade substances.

### 2.2. Collection, Identification, Preparation, and Extraction of the Sample

In May 2024, leaves of *D. racemosus* were gathered from the Lawachara National Park Reserved Forest located in Kamalganj, Moulvibazar, Sylhet, Bangladesh. The Bangladesh National Herbarium, located in Dhaka, Bangladesh, confirmed that the fresh leaves were taken from robust host plants. A voucher specimen (DACB: 124374) has been preserved in the herbarium for future reference. After collecting, the fresh leaves were thoroughly washed with tap water and then rinsed with distilled water to eliminate surface impurities. Subsequently, the leaves were air‐dried in the shade at a controlled temperature of approximately 22°C–24°C for a duration of 7 days. The dehydrated material was ground into a fine powder using a laboratory grinding mill and then filtered through a 40‐mesh sieve. Approximately 450–500 g of the powdered substance was then macerated in 2.5 L of analytical‐grade methanol at room temperature for 15 days, with intermittent shaking and stirring throughout the process. The extract was first filtered with a clean cotton plug and subsequently through Whatman Grade 1 filter paper to remove particulate matter. The combined filtrates were concentrated under reduced pressure using a rotary vacuum evaporator (model no.: DLAB RE 100‐Pro) to create the crude methanolic extract. The extract was then weighed, yielding approximately 25 g (5% yield) and stored in an airtight container at 4°C for future applications.

### 2.3. Experimental Animal Models

The investigation was performed on Swiss albino male mice aged 6–7 weeks, weighing between 25 and 30 g. The mice were sourced from the pharmacy department of Jahangirnagar University in Savar, Dhaka, Bangladesh. They were housed in suitable conditions, which included a 12‐h light–dark cycle, a temperature of 25.0°C±1.0°C, and a relative humidity ranging from 55% to 65%. The subjects were provided with a nutritious diet and had continuous access to water for 24 h. The mice were allowed to adapt to the new laboratory setting for 1 week. The Animal Handling Ethics Committee of Manarat International University sanctioned the studies under permission number MIU/SEST/ERC/2025/001.

### 2.4. GC–MS (Gas Chromatography–Mass Spectrometry) Profiling

For the analysis, a Clarus 690 gas chromatograph, a Clarus SQ 8 C mass spectrometer, and an Elite35 column (30, 0.25, and 0.25 m film) were used. During the 40‐min experiment, helium (99.999%) was used as the carrier gas to continuously inject a 1 *μ*L sample at a flow rate of 1 mL/min. We used electron ionization (EI) at 70 eV. The column oven was programmed to commence at 60°C, ascending to 240°C at a rate of 5°C per minute, followed by a 4‐min hold, without an initial hold period. The intake temperature was maintained at 280°C. The mass spectra were acquired with a scan duration of 1 s over the m/z range of 50–600. Compounds were recognized by associating the results of the mass spectrum with the inbuilt NIST library database [13, 14].

### 2.5. Acute Toxicity Assessment

The median lethal dose (LD50) of the test substance was determined in accordance with OECD Guideline 423 (Organization for Economic Co‐operation and Development). Six mice were allocated to each of the control and treatment groups. Following a 12‐h fast, the treatment group received a single oral gavage dose of MEDR at 5000 mg/kg body weight, whereas the control group received an equivalent volume of normal saline. Animals were monitored continuously for the first 6 h after dosing and then inspected daily for 28 days for clinical signs of toxicity, including aggression, coma, altered locomotor activity, diarrhea, ocular or auditory discharge, labored respiration, anorexia, injury, hypersalivation, lethargy, and mortality [15].

### 2.6. In Vivo Neuropharmacological Study

#### 2.6.1. Experimental Design

Two doses (200 and 400 mg/kg b.w.) were chosen for neuropharmacological testing based on the maximum tolerated dose and the literature (Tareq et al., 2020) to ensure efficacy while minimizing adverse effects. All the treatments, that is, control, standard, and test compound, were given orally. For each in vivo test, 20 male mice were randomly assigned to four groups (*n* = 5), as described by Rahman et al., 2024 [16]:

• Group I (control): distilled water, 10 mL/kg.
• Group II (standard): diazepam, 1 mg/kg.
• Group III (test compound): 200 mg/kg.
• Group IV (test compound): 400 mg/kg.

### 2.7. In Vivo Analysis of Antianxiety Activity

#### 2.7.1. Elevated Plus Maze (EPM) Test

To assess the anxiolytic potential of MEDR, the EPM test was conducted with slight modifications to the standard method of Khalid et al., 2023. The EPM is a plus‐shaped (“+”) apparatus. The maze has a central platform that is 5 × 5 cm, two open arms that are 35 × 5 cm, and two closed arms that are 35 × 5 × 20 cm. The entire apparatus was elevated 25 cm above the ground. After a 30‐min treatment period, each mouse was placed in the middle of the maze and allowed to travel freely. The mice were monitored for 5 min to examine their anxiety‐related behavior by observing the time spent and the number of entries in the open arms and the time spent in the closed arms [17].

#### 2.7.2. Hole‐Board Test

The hole‐board equipment was utilized to examine the anxiolytic properties of MEDR. This device consisted of a wooden board of 25 × 45 × 15 cm, including 16 uniformly distributed holes. The test analyzed the exploratory behavior of mice by monitoring their head‐dipping activity, which acts as a measure of psychological stress or anxiolytic effect. As outlined by [18], Animals were given the treatments control, standard, MEDR 200, and MEDR 400. Thirty minutes after the administration of treatments, each mouse was placed at the center of the apparatus, and their latency of first head dipping was recorded over a 5‐min observation period.

#### 2.7.3. Light–Dark Method

The anxiolytic effect of MEDR was determined using the light–dark test, which is described by Islam et al. [19]. The apparatus used for the light–dark test was derived from the description provided by Crawley and Goodwin (1980). A closed‐top arena (45 × 27 × 27 cm) featuring methacrylate walls and an opaque floor consisted of two compartments. One compartment was painted black (the dark compartment) and the other white (the light compartment). The apparatus was cleaned with cloths moistened with 50% ethanol between each mouse. The compartments were divided by a methacrylate partition featuring a centrally located opening measuring 7.5 × 7.5 cm at floor level. The light–dark compartment was illuminated by a ceiling lamp, providing approximately 350 lux at the floor level of the light–dark apparatus, whereas the black/dark compartment remained unilluminated. Mice were individually positioned in the center of the dark compartment and given 5 min to investigate the activity. The subsequent parameters were assessed: duration in the light and dark compartments.

### 2.8. In Vivo Antidepressant Activity

#### 2.8.1. Forced Swimming Test (FST)

The antidepressant activity of MEDR was determined by using the FST according to Emon et al. [20]. Each animal was separately placed in an open cylindrical glass vessel with a diameter of 20 cm and a height of 50 cm, where 40 cm of the container′s depth was filled with fresh water maintained at a temperature of 25°C±1°C. The test duration was 6 min, comprising 1 min for acclimatization and 5 min to calculate immobility time. An animal was recognized as immobile when it stopped struggling and floated without movement, except for the minimal actions required to keep its head above the water′s surface. A shorter immobility time was considered a sign of antidepressant‐like activity.

### 2.9. In Silico Study

#### 2.9.1. Ligand Structures Preparation for Docking

Based on the results from GC–MS profiling, 20 phytochemical constituents were selected from the MEDR for molecular docking analysis. The three‐dimensional (3D) structures of these compounds were retrieved from the PubChem database in SDF format [21]. The Open Babel GUI software facilitated the conversion of compounds from 2D structures to 3D SDF formats. Prior to docking, energy minimization was performed using Swiss PDB Viewer, considering key parameters such as element type, hybridization, and molecular connectivity. The ligands were then converted into AutoDock‐compatible PDBQT format for further analysis. Diazepam (CID: 3016) was used as a reference ligand to ensure consistency with in vivo experiments, enabling direct comparison between in silico and in vivo results.

#### 2.9.2. Protein Structure Preparation

The PDB format of the 3D crystal structures of the potassium channel receptor (4UUJ), *γ*‐aminobutyric acid Type A (GABA_AA) receptor (6X3X), and hSERT (5I6X) were downloaded from the Protein Data Bank [19, 22]. The potassium channel receptor was chosen from a nonhuman source because its human x‐ray crystallographic structure has not been determined. However, the GABA_AA receptor and serotonin transporter came from humans, so they were good study subjects from a physiological perspective [23]. For molecular docking analysis, we chose chain C of 4UUJ (a voltage‐gated K+ channel), chains D (*α* subunit) and E (*γ* subunit) of 6X3X, and chain A of 5I6X (a sodium‐dependent serotonin transporter). These chains were selected due to their major sites for ligand binding or blocking, which are essential for altering receptor function. [19, 22]. Before docking, all structural components, including nonbonded residues, crystallographic water molecules, and nonessential heteroatoms, were carefully removed to reduce steric interference and improve docking accuracy. After preprocessing with BIOVIA Discovery Studio Visualizer (v21.1.0.20298), only biologically relevant binding domains remained. Using default GROMOS96 force field parameters, Swiss‐PDB Viewer (v4.1.0) minimized structural energy to reduce steric conflicts and optimize receptor model shape [24]. The minimum potential energies for 4UUJ, 6X3X, and 5I6X were −3948.332, −37971.168, and −21058.924 kJ/mol, respectively, indicating relaxed and stable conformations. The improved and optimized PDB structures were employed as receptor models for molecular docking experiments to determine phytochemical ligand binding affinity and interaction.

#### 2.9.3. Molecular Interaction Assessment and Visualization of Ligand–Receptor Complexes

To predict interactions between the chosen ligands and the potassium channel (4UUJ), GABAA_AA receptor (6X3X), and serotonin transporter (5I6X), molecular docking was carried out in PyRx 0.9.8 using AutoDock Vina. The MMFF94 force field was used to minimize the ligands′ energy, and AutoDock Tools was used to convert them into pdbqt format. We used known active‐site residues as a guide when drawing binding pockets. The dimensions of the grid boxes were 15.73 × 14.81 × 31.86 Å (4UUJ), 36.23 × 44.01 × 33.48 Å (6X3X), and 24.03 × 30.34 × 19.05 Å (5I6X) [25]. Negative free energies (kcal/mol) were used to express docking scores; higher binding affinity is correlated with more negative values. Protein ligand complexes were displayed using the BIOVIA Discovery Studio Visualizer v21.1.0 after the highest‐scoring positions were examined for hydrophobic and hydrogen bonding interactions.

### 2.10. Statistical Data Assessment

The data were expressed as the mean ± SEM. One‐way ANOVA was performed using SPSS (Version 25), followed by a post hoc Dunnett′s test to compare each treatment group with the control. A *p* value of less than 0.05 was regarded as statistically significant. Both outcome assessment and data analysis were conducted in a blinded manner, with the analyst unaware of group assignments. All additional calculations and graphing were carried out in Microsoft Excel 2024.

## 3. Results

### 3.1. Phytochemicals From GC–MS

**Figure 1:**
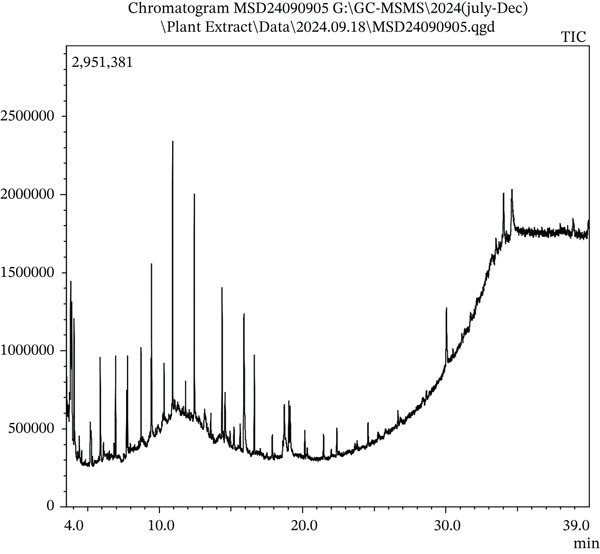
Graphical presentation of GC‐MS findings of the methanolic extract of *Drimycarpus racemosus* Hook. f.

The MEDR was analyzed by GC–MS, identifying 39 phytochemical ingredients (Figure 1and Table 1). Several categories, including aldehydes, fatty acids, esters, terpenoids, phenolics, and hydrocarbons, were identified. The primary constituents detected were hexadecanoic acid methyl ester (8.42%), 3‐methylbenzyl alcohol (4.42%), E4‐nonenal (4.25%), furfural (3.89%), and retinal (3.64%). Multiple bioactive metabolites were identified, including squalene (2.84%), octadecadienoic acid, methyl ester (E, E)‐ (2.04%), and 3‐methylsalicylic acid (1.31%). Fatty acid esters, terpenoids, and phenolic derivatives comprise the largest portion of the chemical profile, and they are recognized for their antioxidant, antibacterial, and anti‐inflammatory properties. The findings offer an extensive phytochemical description of *D. racemosus,* highlighting its significance in ethnomedicine and prospective pharmaceutical applications.

**Table 1:** Phytochemicals found in *Drimycarpus racemosus* Hook. f. through GC–MS.

s	R. time	Area %	Compound name
1	3.53	4.25	4‐Nonenal, (E)‐
2	3.652	3.29	3,3‐Dimethoxy‐2‐butanone
3	3.813	8.29	Furfural
4	3.895	4.42	3‐Methylbenzyl alcohol
5	4.055	3.64	Retinal
6	6.84	0.38	Vanillic acid
7	8.901	0.40	D‐verbenone
8	11.074	1.28	Tridecanoic acid, 4,8,12‐trimethyl‐, methyl ester
9	11.21	0.63	4‐Methylthiane, S‐oxide
10	11.273	0.55	7‐Methyldecanoic acid
11	14.13	0.41	Bicyclo [3.1.1] heptan‐3‐ol, 6,6‐dimethyl‐2‐methylene‐, [1S‐(1. alpha.,3. alpha.,5. alpha.)]‐
12	14.23	0.41	(1R,2S,4R)‐2,7,7‐Trimethylbicyclo [2.2.1] heptan‐2‐ol
13	14.379	4.00	2,6‐Dihydroxybenzoic acid
14	14.47	0.44	1,3,5‐Cycloheptatriene, 7‐ethyl‐
15	14.585	2.03	Neophytadiene
16	15.211	0.86	3,7,11,15‐Tetramethyl‐2‐hexadecen‐1‐ol
17	15.906	7.28	Hexadecanoic acid, methyl ester
18	16.87	0.37	Decahydronaphthalene‐2‐carboxylic acid
19	18.62	0.61	9,11‐Octadecadienoic acid, methyl ester, (E, E)‐
20	18.723	2.70	7‐Hexadecenoic acid, methyl ester, (Z)‐
21	18.84	0.83	8‐Methylenecyclooctene‐3,4‐diol
22	19.124	2.05	Methyl stearate
23	27.635	0.42	4‐Hydroxybenzoic acid
24	30.042	2.84	Squalene
25	31.112	0.40	5‐(7a‐Isopropenyl‐4,5‐dimethyl‐octahydroinden‐4‐yl)‐3‐methyl‐pent‐2‐enal
26	32.47	0.57	Fumaric acid
27	32.865	0.82	4H‐Cyclopropa [5’,6’] benz [1’,2’:7,8] azulene[5,6‐b] oxiren‐4‐one, 8,8a‐bis(acetyloxy)‐2a‐[(acetyloxy)methyl]‐1,1a,1b,1c,2a,3,3a,6a
28	32.955	0.80	1,7‐Dioxadispiro [4.0.5.3] tetradec‐12‐ene‐11,14‐dione, 12‐hydroxy‐2,2,8,8‐tetramethyl‐13‐(3‐methyl‐1‐oxobutyl)‐
29	33.065	0.91	Dodecahydropyrido[1,2‐b] isoquinolin‐6‐one
30	33.125	0.63	Trans‐3‐Ethoxycarbonyloxy‐4‐methoxycinnamic acid
31	33.165	0.79	[5,5‐Dimethyl‐6‐(3‐methyl‐buta‐1,3‐dienyl)‐7‐oxa‐bicyclo [4.1.0] hept‐1‐yl]‐methanol
32	33.223	1.20	9H‐Fluorene‐2‐carboxylic acid, 9‐oxo‐, (2‐hydroxyethyl) (methyl)amide
33	33.526	0.67	4 (15)‐Selinene‐11,12‐diol
34	33.645	0.74	Benzoic acid,
35	33.832	0.46	Silicic acid
36	33.93	1.31	3‐Methylsalicylic acid
37	34.03	3.64	Beta‐sitosterol acetate
38	34.612	3.03	Alpha‐tocopheryl acetate
39	38.873	0.83	Feselol

Abbreviation: R. time, retention time.

### 3.2. Acute Toxicity Evaluation in Animal Models

The administration of a single, high dose of 5000 mg/kg b.w. of MEDR produced no mortality, clinical signs of toxicity, or abnormal behavior during the 28‐day observation period, supporting the safety margin of the two doses selected for the in vivo study (200 and 400 mg/kg b.w.).

### 3.3. In Vivo Anxiolytic Potential Assessment

#### 3.3.1. EPM Test

Compared to control (74.2 ± 2.71 s; 10.4 ± 1.57 entries), diazepam significantly increased open‐arm time (191.2 ± 4.71 s) and entries (20.8 ± 1.74) while reducing closed‐arm time (108.8 ± 4.71 s) (∗*p* < 0.05). MEDR at 200 mg/kg also increased open‐arm time (93.8 ± 3.06 s) and entries (11.0 ± 0.95) with reduced closed‐arm time (206.2 ± 3.06 s), whereas 400 mg/kg produced a stronger effect (140.0 ± 1.79 s; 17.2 ± 1.43 entries; closed arm: 160.0 ± 1.79 s) (∗*p* < 0.05). These results indicate a dose‐dependent anxiolytic effect of MEDR (Table 2).

**Table 2:** Effects of the methanolic extract of *Drimycarpus racemosus* Hook. f. in the elevated plus maze test.

Animal group	Time spent in open arm (sec)	Time spent in close arm (sec)	No of entries in the open arm
Control	74.2 ± 2.71	225.8 ± 2.71	10.4 ± 1.57
Diazepam	191.2 ± 4.71	108.8 ± 4.71	20.8 ± 1.74
MEDR 200	93.8 ± 3.06	206.2 ± 3.06	11.0 ± 0.95
MEDR 400	140.0 ± 1.79^∗^	160.0 ± 1.79^∗^	17.2 ± 1.43^∗^

*Note:* Here, Mean ± SEM (*n* = 5) is used to display the values.

^∗^ *p* < 0.05 compared to control (Dunnett′s test).

### 3.4. Hole‐Board Test

Compared to control (33.0 ± 1.82; 15.4 ± 1.63 *s*), diazepam significantly increased the number of head dips (65.4 ± 2.20) and reduced the latency to first head dipping (4.4 ± 0.51 *s*) (∗*p* < 0.05). MEDR at 200 mg/kg showed a slight increase in head dipping (32.4 ± 2.01) with reduced latency (12.6 ± 0.93 s), whereas 400 mg/kg produced a more pronounced effect (65.6 ± 2.29; 7.8 ± 0.58 *s*) (∗*p* < 0.05). These results indicate an anxiolytic effect of MEDR, with a stronger response at 400 mg/kg (Table 3).

**Table 3:** Effects of the methanolic extract of *Drimycarpus racemosus* Hook. f. in the hole‐board test.

Animal group	No of head dipping	Latency to the first head dipping (sec)
Control	33.0 ± 1.82	15.4 ± 1.63
Diazepam	65.4 ± 2.20	4.4 ± 0.51
MEDR 200	32.4 ± 2.01	12.6 ± 0.93
MEDR 400	65.6 ± 2.29^∗^	7.8 ± 0.58

*Note:* Here, Mean ± SEM (*n* = 5) is used to display the values.

^∗^ *p* < 0.05 compared to control (Dunnett′s test).

### 3.5. Light and Dark Test

Compared to control (92.4 ± 1.36 *s*; 208.4 ± 4.39 *s*), diazepam significantly increased time spent in the light box (140.14 ± 1.00 *s*) and decreased time in the dark box (158.6 ± 3.71 *s*) (∗*p* < 0.05). MEDR at 200 mg/kg showed a moderate increase in light box time (112.2±2.27 s) with reduced dark box time (185.6 ± 5.86 *s*), whereas 400 mg/kg produced a stronger effect (137.4 ± 1.33 *s*; 166.0 ± 3.74 *s*) (∗*p* < 0.05). These results indicate a dose‐dependent anxiolytic effect of MEDR (Table 4).

**Table 4:** Effects of the methanolic extract of *Drimycarpus racemosus* Hook. f. in the light and dark test.

Animal group	Time spent in light box (sec)	Time spent in dark box (sec)
Control	92.4 ± 1.36	208.4 ± 4.39
Diazepam	140.143 ± 1.00	158.6 ± 3.71
MEDR 200	112.2 ± 2.27	185.6 ± 5.86
MEDR 400	137.4 ± 1.33^∗^	166.0 ± 3.74^∗^

*Note:* Here, Mean ± SEM (*n* = 5) is used to display the values.

^∗^ *p* < 0.05 compared to control (Dunnett′s test).

### 3.6. Antidepressant Activity Study

#### 3.6.1. FST

Compared to control (182.6 ± 1.94 s), diazepam significantly reduced immobility time (94.4 ± 1.50 s) (∗*p* < 0.05). MEDR at 200 mg/kg also reduced immobility time (122.0 ± 1.38 s), while 400 mg/kg produced a greater reduction (102.2 ± 1.16 s) (∗*p* < 0.05). These results suggest a dose‐dependent antidepressant‐like effect of MEDR (Table 5).

**Table 5:** Effects of the methanolic extract of *Drimycarpus racemosus* Hook. f. in the forced swimming test.

Animal group	Immobility time (sec)
Control	182.6 ± 1.94
Diazepam	94.4 ± 1.50
MEDR 200	122 ± 1.38
MEDR 400	102.2 ± 1.16^∗^

*Note:* Here, Mean ± SEM (*n* = 5) is used to display the values.

^∗^ *p* < 0.05 compared to control (Dunnett′s test).

### 3.7. In Silico Study

**Figure 2:**
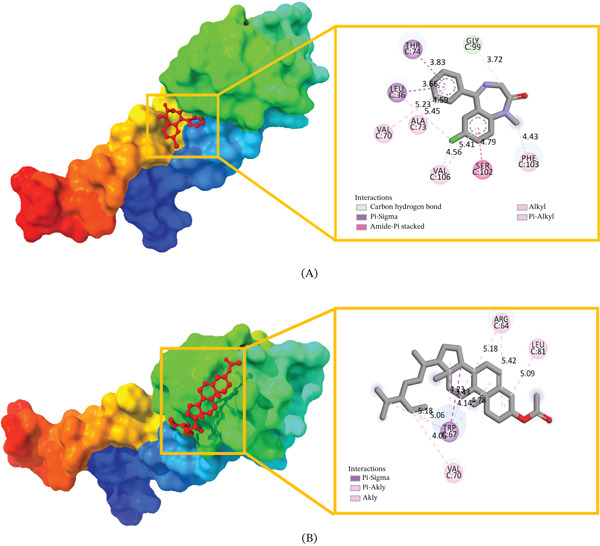
(A) 2D and 3D graphical visualization showing the binding interactions of diazepam and *β*‐sitosterol acetate with the active site of the potassium channel receptor (PDB ID: 4UUJ). (B) 2D and 3D graphical visualization showing the binding interactions of *β*‐Sitosterol acetate with the active site of the potassium channel receptor (PDB ID: 4UUJ).

**Figure 3:**
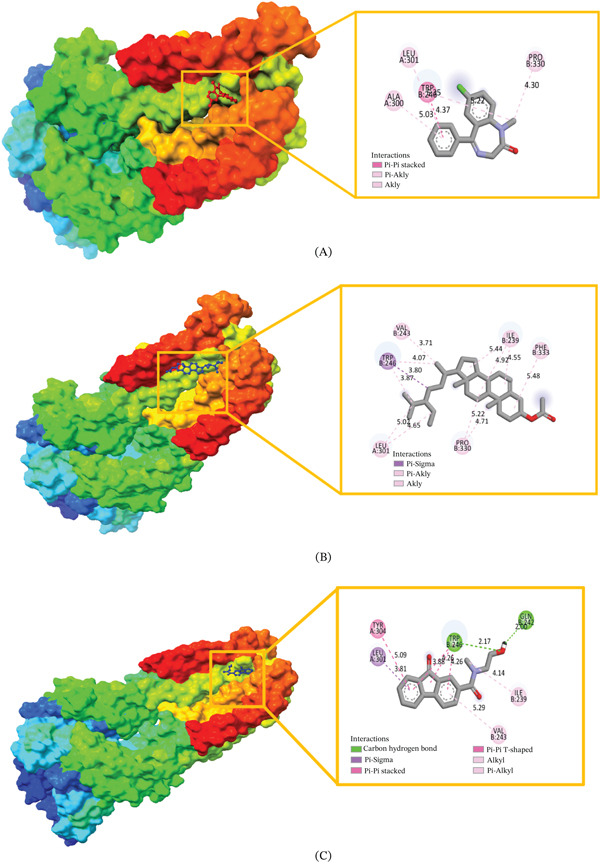
(A) 2D and 3D graphical visualization showing the binding interactions of diazepam with the active site of the GABAA (6X3X) receptor. (B) 2D and 3D graphical visualization showing the binding interactions of beta‐sitosterol acetate with the active site of the GABAA (6X3X) receptor. (C) 2D and 3D graphical visualization showing the binding interactions of 9H‐fluorene‐2‐carboxylic acid, 9‐oxo‐, (2‐hydroxyethyl) (methyl) amide with the active site of the GABAA (6X3X) receptor.

**Figure 4:**
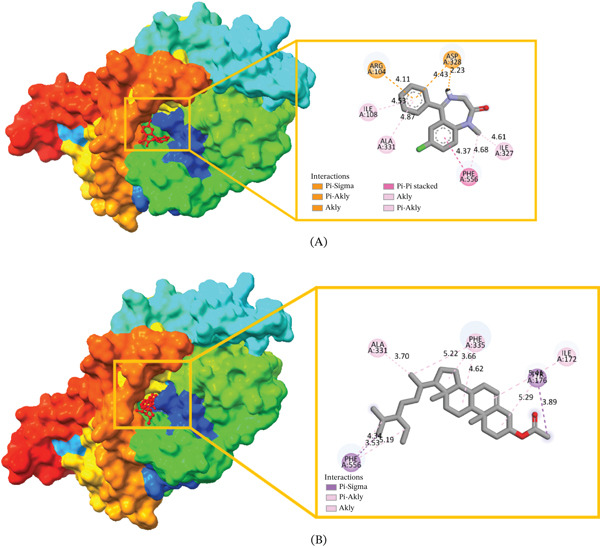
(A) 2D and 3D graphical visualization showing the binding interactions of diazepam with the active site of serotonin (5I6X) receptor. (B) 2D and 3D graphical visualization showing the binding interactions of beta‐sitosterol acetate with the active site of serotonin (5I6X) receptor.

Molecular docking of MEDR‐derived ligands and diazepam with potassium channel receptor (PDB ID: 4UUJ), GABA receptor (PDB ID: 6X3X), and hSERT (PDB ID: 5I6X) have been shown in Tables 6, 7 and 8 and Figures 2, 3 and 4, respectively. Molecular docking revealed that all assessed ligands interact through diverse noncovalent interactions. Two principal components of *D. racemosus* had greater affinities than the reference medication diazepam.

**Table 6:** Molecular docking analysis of diazepam and selected ligands with the potassium channel (PDB ID: 4UUJ).

Sl. no.	Compound name	Binding affinity of 4UUJ (kcal/mol)	Hydrogen bond interactions	Hydrophobic interactions
STD	Diazepam	−6.9	GLY99	LEU36, THR74, SER102, PHE103, LEU36, VAL106, VAL70, ALA73
1	Feselol	−7.7	ALA50	LEU46, TRP87, VAL91, VAL94
2	Beta‐sitosterol acetate	−7.0	N/A	TYR62, ALA42, LEU46, LEU66, TYR45
3	Alpha‐tocopheryl acetate	−6.6	N/A	TRP87, VAL39, ALA42, LEU46, VAL91, VAL94, VAL95
4	1,7‐Dioxadispiro [4.0.5.3] tetradec‐12‐ene‐11,14‐dione, 12‐hydroxy‐2,2,8,8‐tetramethyl‐13‐(3‐methyl‐1‐oxobutyl)‐	−6.5	GLY77, PRO83	TYR82, MET96
5	9H‐fluorene‐2‐carboxylic acid, 9‐oxo‐, (2‐hydroxyethyl) (methyl)amide	−6.5	TRP68, GLU71, THR72	TYR82
6	Retinal	−6.2	N/A	LEU36, ILE100, PHE103

**Table 7:** Binding affinities and interaction profiles of diazepam and selected ligands against GABA receptor (PDB ID: 6X3X).

SI. no.	Compound name	Binding affinity of 6X3X (kcal/mol)	Hydrogen bond interactions	Hydrophobic interactions
STD	Diazepam	−6.7	N/A	TRP246, ALA300, LEU301
1	Foselol	−8.7	SER299	LEU332, PHE329, PHE296, ALA328
2	Beta‐sitosterol acetate	−8.2	N/A	LEU301, LEU301, LEU301, TYR304, TYR304
3	9H‐Fluorene‐2‐carboxylic acid, 9‐oxo‐, (2‐ hydroxyethyl) (methyl)amide	−8.1	TRP246, GLN242	TRP246, VAL243
4	Alpha‐tocopheryl acetate	−8.1	ILE228	MET286, ILE228, PRO233, PHE289
5	1,7‐Dioxadispiro [4.0.5.3] tetradec‐12‐ene‐11,14‐dione, 12‐hydroxy‐2,2,8,8‐tetramethyl‐13‐(3‐methyl‐1‐oxobutyl)‐	−7.4	TRP246	TRP246, LEU301, TYR304
6	4 (15)‐Selinene‐11,12‐diol	−7.2	GLN309	LEU297, ALA300, LEU301, VAL243, TYR304, TRP246

**Table 8:** Binding affinities and interaction profiles of the diazepam and selected ligands against the human serotonin transporter (PDB ID: 5I6X).

SI. no.	Compound name	Binding affinity of 5I6X (kcal/mol)	Hydrogen bond interactions	Hydrophobic interactions
STD	Diazepam	−7.4	ASP328	PHE556, ILE108, ALA331
1	Foselol	−11.0	PHE335	ILE172, PHE335, VAL501
2	Beta‐sitosterol acetate	−9.9	N/A	PHE556, TYR176, ALA331, ILE327, PHE335
3	Squalene	−9.4	N/A	PHE341, TYR579, PRO560, PRO561, ILE172, PRO499, PHE335, TYR495, PHE556
4	Retinal	−8.7	ARG104	ILE172, PHE335, PHE341
5	9H‐fluorene‐2‐carboxylic acid, 9‐oxo‐, (2‐hydroxyethyl) (methyl)amide	−8.4	TYR175, GLY498, PHE335	PHE335, ALA331
6	Dodecahydropyrido[1,2‐b] isoquinolin‐6‐one	−8.1	N/A	ILE172, VAL501, TYR176, PHE341

## 4. Discussion

Herbal medicines play a crucial role in primary healthcare, especially in developing countries [26]. Researchers are working to collect and preserve unpublished data on medicinal herbs used by various ethnic groups to improve future healthcare. The scientific validation of traditional therapies is vital to developing effective pharmaceuticals and treatment methodologies, yet it remains insufficient [17]. Additional studies are required to determine the effectiveness and safety of these herbal products. However, studies indicate that medicinal plants contain multiple bioactive compounds with diverse pharmacological effects and serve as adjunctive therapies for chronic diseases and neurological disorders [27]. It has been demonstrated that several herbs, such as *B. monnieri*, *L. angustifolia*, *and C. sativus*, exhibit anxiolytic and antidepressant effects that are comparable to those of conventional pharmaceuticals.

*D. racemosus* belongs to the Anacardiaceae family, which comprises well‐known species such as *M. indica*, *S. lentiscifolius*, and *P. microcarpa,* that have been proven to exert anxiolytic‐ and antidepressant‐like effects through modulation of key neurotransmitter systems, including serotonergic, dopaminergic, and GABAergic pathways. These pharmacological activities are consistently validated through standard behavioral paradigms, including the forced swim test, tail suspension test, and EPM, often without inducing notable sedative effects. This study demonstrated the anxiolytic and antidepressant properties of the methanolic extract of *D. racemosus* leaves using well‐established animal models. The research aimed to elucidate the multitarget potential of the extract through a series of in vivo and in silico assays.

GC–MS analysis of *D. racemosus* identified 39 phytochemical compounds, which include fatty acids, esters, long‐chain aldehydes and ketones, phenolic derivatives, terpenoids, and hydrocarbons. The study identified a variety of bioactive metabolites, such as hexadecanoic acid methyl ester (8.42%), 3‐methylbenzyl alcohol TBDMS derivative (4.42%), E4‐nonenal (4.25%), furfural (3.89%), retinal (3.64%), squalene (2.84%), octadecadienoic acid, methyl ester (E, E)‐ (2.04%), and 3‐methylsalicylic acid (1.31%) [14]. The varied chemical composition of *D. racemosus* reveals numerous bioactive compounds, indicating possible pharmacological advantages. Fatty acid esters, terpenoids, and phenolic derivatives constitute the predominant components of the chemical profile, noted for their antioxidant, antibacterial, and anti‐inflammatory properties [28].

Beyond single‐target medications, complex natural products often modulate multiple signaling pathways simultaneously. Saffron‐derived crocin has significant anti‐inflammatory and anticancer effects by synergistically downregulating COX‐2 and associated signaling pathways [3]. Plant‐derived essential oils and monoterpenes, including limonene and linalool, have shown to provide synergistic antioxidant and anxiolytic effects by regulating the Nrf2/ARE redox axis and suppressing the inflammatory cascade. These findings provide a solid basis for future research into its therapeutic and dietary applications. The methanolic leaf extract of *D. racemosus* exhibited a clear dose‐dependent anxiolytic effect across all behavioral assessments. The EPM test revealed that doses of 200 and 400 mg/kg significantly increased the time spent in open arms and the number of entries into open arms while concurrently reducing the time spent in closed arms; the significant increase in open‐arm exploration and decreased time spent in closed arms suggest a reduction in anxiety‐related behavior [29]. The increased dosage of 400 mg/kg produced effects comparable to those of diazepam, the standard anxiolytic, thereby confirming its potency. The hole‐board test supported these findings, showing that MEDR at 400 mg/kg significantly increased head‐dip frequency, which indicates enhanced exploration motivation and reduced emotional restraint [30]. In the light–dark test, the extract markedly prolonged the duration in the illuminated compartment, indicating diminished light aversion and corroborating its anxiolytic‐like effectiveness [31]. The lack of a notable effect at the lower dosage, 200 mg/kg suggests that the anxiolytic response is dose‐dependent and may entail cumulative modulation at the receptor level [32]. All mice displayed uniform behavioral responses, indicating that MEDR operates via mechanisms analogous to those of benzodiazepines, likely involving GABAergic modulation [33]. The extract′s bioactive phytoconstituents, such as flavonoids, terpenoids, and phenolic compounds, which are known for their neuroprotective qualities and interaction with GABAA receptors, may be responsible for the reported results [34]. The antidepressant activity was assessed using the FST, which demonstrated that MEDR significantly decreased immobility time in a dose‐dependent manner relative to the control group. The 400 mg/kg dose showed a more pronounced effect than the 200 mg/kg dose, with less immobility time, which indicates a dose‐dependent effect. Lastly, the dose of 400 mg/kg exhibited the same response as diazepam, which confirms strong antidepressant‐like activity [35]. These behavioral findings align with the in silico results, suggesting serotonergic or GABAergic pathway involvement.

This study conducts a comprehensive analysis of the anxiolytic and antidepressant effects linked to three principal protein targets, that is, potassium channels, GABAA receptors, and the hSERT, via molecular docking assessments. These receptors are essential because inhibitory neurotransmission regulates the firing of neurons and has an effect on the serotonin systems that are responsible for emotional health. They also play a vital role in neuropharmacology, mediating inhibitory neurotransmission, influencing neuronal excitability, and regulating mood and anxiety through serotonergic pathways [36]. As we gain more knowledge about these pathways, it becomes abundantly clear that blocking those receptors can improve treatments for mental health and can also improve the lives of a significant number of people. The potassium channel receptor (PDB ID: 4UUJ) is involved in changing how neurons depolarize. When ligands bind to this receptor, they can boost inhibitory signaling, which leads to sedative and anxiolytic effects. In this study, two compounds—2‐[4‐cyclohexylbutanoylamino]‐3‐chloro‐1,4‐naphthoquinone and *β*‐sitosterol acetate—derived from the *D. racemosus* extract exhibited high binding affinities of −7.2 and −7.0 kcal/mol, respectively. These values are higher than those of the standard anxiolytic diazepam (−6.9 kcal/mol). Furthermore, multiple hydrophobic and hydrogen interactions with residues TYR45, LEU66, and TRP67 suggest channel stabilization, which indicates a potassium channel modulated antidepressant like mechanism [37].

Furthermore, six phytochemicals of MEDR showed stronger interactions with the GABAA receptor, with docking scores ranging from −8.2 to −6.8 kcal/mol, which also exceed the score for diazepam (−6.7 kcal/mol). Beta‐sitosterol acetate and 9 H‐fluorene‐2‐carboxylic acid, 9‐oxo‐, (2‐hydroxyethyl) (methyl) amide showed various hydrophobic and *π*–*π* interactions with residues such as TRP246, LEU301, and PRO330, in addition to hydrogen bonding with GLN242 and TRW246 (Figure 3B,C). The key interactions relate to the benzodiazepine‐binding pocket of GABA. The receptor enhances GABA‐mediated chloride influx and promotes neuronal hyperpolarization. The increased affinity and stabilization of this receptor conformation suggest that *D. racemosus* metabolites may produce anxiolytic and sedative effects through positive allosteric modulation akin to benzodiazepines [38, 39].

Moreover, six compounds exhibited exceptional binding affinities for the hSERT, with docking energies ranging from −9.9 to −7.5 kcal/mol, exceeding the standard diazepam (−7.4 kcal/mol). Among them, beta‐sitosterol acetate and 2‐[4‐cyclohexylbutanoylamino]‐3‐chloro‐1,4‐naphthoquinone exhibited higher binding affinities at −9.9 and −9.4 kcal/mol, respectively. They formed strong hydrogen bonds with THR497 and ASP98, combined with hydrophobic and *π*–*π* interactions at PHE556 and TYR176, suggesting an effective suppression of serotonin reuptake (Figure 4A,B). These findings indicate that the extract may have dual therapeutic potential in the treatment of both anxiety and depressive disorders, likely through the synergistic modulation of serotonergic and GABAergic systems, as the inhibition of hSERT is recognized as a confirmed mechanism for antidepressant efficacy [40].

In conclusion, both in vivo and in silico analyses confirmed the anxiolytic and antidepressant potential of the methanolic leaf extract of *D. racemosus*. The extract elicited notable behavioral responses suggestive of GABAergic and serotonergic modulation. Molecular docking analysis revealed that the key phytoconstituents *β*‐sitosterol acetate and 2‐[4‐cyclohexylbutanoylamino]‐3‐chloro‐1,4‐naphthoquinone exhibited greater binding affinities for the potassium channel, GABAA receptor, and hSERT compared with diazepam. Consequently, their synergistic effects may enhance the treatment of individuals with these mental health disorders. The multireceptor interactions indicate a synergistic neuropharmacological mechanism that underpins the extract′s central nervous system activity. *D. racemosus* presents as a viable, plant‐derived candidate for the formulation of safe therapeutics targeting anxiety and depression, necessitating additional research on the isolation of active compounds and receptor‐level validation.

## 5. Conclusion

The present study provides supportive in vivo and in silico evidence for the anxiolytic and antidepressant potential of the methanolic leaf extract of *D. racemosus*. Behavioral experiments demonstrated dose‐dependent anxiolytic and antidepressant‐like effects, comparable to diazepam, possibly through modulation of GABAergic and serotonergic pathways. GC–MS analysis revealed a diverse phytochemical profile enriched with fatty acids, terpenoids, and phenolic compounds, which may contribute to the observed neuropharmacological activities. Molecular docking analyses further suggested that certain constituents, particularly *β*‐sitosterol acetate and 2‐[4‐cyclohexylbutanoylamino]‐3‐chloro‐1,4‐naphthoquinone, possess favorable binding affinities toward the potassium channel, GABAA receptor, and hSERT when compared with the reference ligand diazepam. However, these computational findings are predictive in nature and do not constitute direct evidence of receptor binding or target engagement. Therefore, the proposed multitarget interactions should be interpreted cautiously until confirmed through experimental validation using biophysical and molecular approaches such as surface plasmon resonance (SPR), isothermal titration calorimetry (ITC), enzymatic assays, and receptor‐level studies. Overall, the findings suggest that *D. racemosus* may represent a promising source of bioactive compounds for future neuropharmacological research and the development of plant‐based therapeutic candidates for anxiety and depressive disorders. Further studies involving isolation of active constituents, mechanistic investigations, neurochemical analyses, and experimental target validation are necessary to clarify its precise molecular mechanisms and translational potential in clinical neuropsychopharmacology.

**Nomenclature**
DMSO: dimethyl sulfoxide
EPM: elevated plus maze
GABA_A_
: gamma‐aminobutyric acid Type A receptor
GC‐MS: gas chromatography–mass spectrometry
MEDR: methanolic extract of *Drimycarpus racemosus*
NIST: National Institute of Standards and Technology
PDB: Protein Data Bank

## Author Contributions

Md. Rakibul Islam: investigation and software. Md. Sajib Ali: investigation, data curation, data analysis, formal analysis, and writing. Priota Islam Meem: original writing. M. A. Rafi, Sanjana Haque Esha, and Md. Mehedi Hasan: investigation, data curation, and software. Abdullah Ripon: data curation, writing, and editing. Payar Hossain and Nilufar Sultana: conceptualization, supervision, investigation, data curation, data analysis, software, writing, and editing.

## Funding

No funding was received for this manuscript.

## Ethics Statement

The Institutional Animal Ethics Committee of the Department of Pharmacy, Faculty of Science, Engineering and Technology, Manarat International University, Bangladesh authorized animal experiments used in the study. All authors declare that the “Principles of Laboratory Animal Care” (NIH publication No. 85‐23, revised 1985) and any relevant national regulations were followed. All experiments, including the investigation of the plant under study, were reviewed and approved by Manarat International University′s ethical committee (approval number MIU/SEST/ERC/2025/001).

## Conflicts of Interest

The authors declare no conflicts of interest.

## Data Availability

The data that support the findings of this study are available from the corresponding author upon reasonable request.

## References

[1] Faruk M. , Ching U. , and Chowdhury K. , Mental Health and Well-Being of Indigenous People During the COVID-19 Pandemic in Bangladesh, Heliyon. (2021) 7, no. 7, e07582, 10.1016/j.heliyon.2021.e07582, 34345744. 34345744 PMC8319571

[2] Mora S. , Díaz-Véliz G. , Millán R. , Lungenstrass H. , Quirós S. , Coto-Morales T. , and Hellión-Ibarrola M. C. , Anxiolytic and Antidepressant-Like Effects of the Hydroalcoholic Extract From Aloysia polystachya in Rats, Pharmacology, Biochemistry, and Behavior. (2005) 82, no. 2, 373–378, 10.1016/j.pbb.2005.09.007, 16278011. 16278011

[3] Awad B. , Hamza A. A. , Al-Maktoum A. , Al-Salam S. , and Amin A. , Combining Crocin and Sorafenib Improves their Tumor-Inhibiting Effects in a Rat Model of Diethylnitrosamine-Induced Cirrhotic-Hepatocellular Carcinoma, Cancers. (2023) 15, no. 16, 10.3390/cancers15164063. PMC10452334 37627094

[4] Kasper S. , Boer J. A. , and Sitsen J. M. A. , Handbook of Depression and Anxiety: A Biological Approach, Second Edition, 2003, 2nd edition, CRC Press, 10.3109/9780203911822.

[5] Rahman M. A. , Sarkar K. K. , Aktaruzzaman M. , Mitra T. , Sarker M. T. , Abid M. A. , Mazumder K. , Barman A. K. , Molla N. U. , and Hossain A. S. M. M. A. , Evaluation of Antioxidant, Anxiolytic and Antidepressant Potential of Saurauia roxburghii Wall. Leaves: Supported by In Vitro, In Vivo, and In Silico Approaches, Phytomedicine Plus. (2025) 5, no. 2, 100792, 10.1016/j.phyplu.2025.100792.

[6] García Ríos R. , Mora-Perez A. , Ramos-Molina A. , and Soria Fregozo C. , Neuropharmacology of Secondary Metabolites from Plants With Anxiolytic and Antidepressant Properties, Behavioral Pharmacology-From Basic to Clinical Research, 2020, IntechOpen, 10.5772/intechopen.90919.

[7] Chirumamilla P. and Taduri S. , Assessment of In Vitro Anti-Inflammatory, Antioxidant and Antidiabetic Activities of *Solanum khasianum* Clarke, Vegetos. (2023) 36, no. 2, 575–582, 10.1007/s42535-022-00410-6.

[8] Ozsavci D. , Ozakpinar O. B. , Cetin M. , and Aricioglu F. , Level of Clinical Evidence of Herbal Complementary Therapies in Psychiatric Disorders, Psychiatry and Clinical Psychopharmacology. (2019) 29, no. 3, 239–243, 10.1080/24750573.2019.1625587.

[9] Hong R. , Zhang Z. , Sun X. , Chen Y. , Wei H. L. , Chen Q. , Zhou L. , Zhou X. , Chen W. , and Wan J. , A Comprehensive Pharmacological Review of Natural Products Active Ingredients in Ulcerative Colitis, Natural Products Journal. (2025) 16, no. 2, e22103155348415, 10.2174/0122103155348415241119073736.

[10] Aruoma O. I. , Bahorun T. , and Jen L.-S. , Neuroprotection by Bioactive Components in Medicinal and Food Plant Extracts, Mutation Research/Reviews in Mutation Research. (2003) 544, no. 2–3, 203–215, 10.1016/j.mrrev.2003.06.017. 14644322

[11] Kirtikar K. R. and Basu B. D. , Indian Medicinal Plants, 1999, 3, International Book Distributors Book Sellers and Publishers, https://www.scirp.org/reference/referencespapers?referenceid=1411861.

[12] Rahman M. A. , Plant Diversity in Hazarikhil Wildlife Sanctuary of Chittagong and Its Conservation Management, Journal of Biodiversity Conservation and Bioresource Management. (2018) 3, no. 2, 43–56, 10.3329/jbcbm.v3i2.36027.

[13] Shelke D. B. , Tayade S. , Gawande P. , and Sonawane H. B. , GC-MS Analysis and Antioxidant Potential of Wild Underutilized Medicinally Important Legume, Velvet Bean (Mucuna Pruriens L. DC.), Notulae Scientia Biologicae. (2022) 14, no. 1, https://www.notulaebiologicae.ro/index.php/nsb/article/view/11098.

[14] Ali M. S. , Islam M. R. , Rafi M. A. , Esha S. H. , Hasan M. M. , Hossain P. , Ripon A. , and Sultana N. , Integrative GC–MS, FT-IR, and Molecular Docking Analysis of Drimycarpus racemosus (Roxb.) Hook.f ex Marchand: In Vivo Analgesic and Antidiabetic Potential, Food Science & Nutrition. (2026) 14, no. 8, e72204, 10.1002/fsn3.72204, 42548790. 42548790 PMC13429936

[15] Kundu N. , Chakrabarty B. , Acharyya R. , Sarker M. M. R. , Mazid M. , and Rahman M. , Phytochemical Screening, Analgesic, Anti-Hyperglycemic and Hepatoprotective Potentials of Codariocalyx motorius (Houtt.) Leaves Extract, Journal of Medicinal Plants Studies. (2024) 12, no. 1, 63–69, 10.22271/plants.2024.v12.i1a.1627.

[16] Rahman R. , Siddique T. , Nipa F. A. , Sultana S. , Devi P. , Islam F. , Nainu F. , Obaidullah A. J. , Emran T. B. , and Khatun M. R. , Bark Extract of Chaetocarpus castanocarpus (Roxb.) Exhibits Potent Sedative, Anxiolytic, and Antidepressant Effects Through an In Vivo Approach in Swiss Albino Mice, European Review for Medical & Pharmacological Sciences. (2024) 28, no. 3, https://www.researchgate.net/publication/378206158_Bark_extract_of_Chaetocarpus_castanocarpus_Roxb_exhibits_potent_sedative_anxiolytic_and_antidepressant_effects_through_an_in_vivo_approach_in_Swiss_albino_mice. 10.26355/eurrev_202402_35359 38375725

[17] Khalid A. A. , Jabeen Q. , and Javaid F. , Anxiolytic and Antidepressant Potential of Methanolic Extract of *Neurada procumbens* Linn. in Mice, Dose-Response. (2023) 21, no. 2, 15593258231169584, 10.1177/15593258231169584, 37063345. 37063345 PMC10102953

[18] Rakib A. , Ahmed S. , Islam M. A. , Uddin M. M. N. , Paul A. , Chy M. N. U. , Emran T. B. , and Seidel V. , Pharmacological Studies on the Antinociceptive, Anxiolytic and Antidepressant Activity of Tinospora crispa, Phytotherapy Research. (2020) 34, no. 11, 2978–2984, 10.1002/ptr.6725, 32430999. 32430999

[19] Islam M. S. , Hossain R. , Ahmed T. , Rahaman M. M. , al-Khafaji K. , Khan R. A. , Sarkar C. , Bappi M. H. , de Andrade E. M. , Araújo I. M. , Coutinho H. D. M. , Kowalska G. , Kowalski R. , Hanif M. A. , and Islam M. T. , Anxiolytic-Like Effect of Quercetin Possibly Through GABA Receptor Interaction Pathway: In Vivo and In Silico Studies, Molecules. (2022) 27, no. 21, 10.3390/molecules27217149, 36363979. PMC9656213 36363979

[20] Emon N. U. , Alam S. , Rudra S. , Riya S. R. , Paul A. , Hossen S. M. M. , Kulsum U. , and Ganguly A. , Antidepressant, Anxiolytic, Antipyretic, and Thrombolytic Profiling of Methanol Extract of the Aerial Part of Piper nigrum: In Vivo, In Vitro, and In Silico Approaches, Food Science & Nutrition. (2021) 9, no. 2, 833–846, 10.1002/fsn3.2047, 33598167. 33598167 PMC7866625

[21] Gervasoni S. , Malloci G. , Bosin A. , Vargiu A. V. , Zgurskaya H. I. , and Ruggerone P. , AB-DB: Force-Field Parameters, MD Trajectories, QM-Based Data, and Descriptors of Antimicrobials, Scientific Data. (2022) 9, no. 1, 10.1038/s41597-022-01261-1, 35365662. PMC8976083 35365662

[22] Lenaeus M. J. , Burdette D. , Wagner T. , Focia P. J. , and Gross A. , Structures of KcsA in Complex With Symmetrical Quaternary Ammonium Compounds Reveal a Hydrophobic Binding Site, Biochemistry. (2014) 53, no. 32, 5365–5373, 10.1021/bi500525s, 25093676. 25093676 PMC4139162

[23] Xia G. , Han Y. , Meng F. , He Y. , Srisai D. , Farias M. , Dang M. , Palmiter R. D. , Xu Y. , and Wu Q. , Reciprocal Control of Obesity and Anxiety-Depressive Disorder via a GABA and Serotonin Neural Circuit, Molecular Psychiatry. (2021) 26, no. 7, 2837–2853, 10.1038/s41380-021-01053-w, 33767348. 33767348 PMC8505263

[24] Woo H. , Park S. J. , Choi Y. K. , Park T. , Tanveer M. , Cao Y. , Kern N. R. , Lee J. , Yeom M. S. , Croll T. I. , Seok C. , and Im W. , Developing a Fully Glycosylated Full-Length SARS-CoV-2 Spike Protein Model in a Viral Membrane, Journal of Physical Chemistry B. (2020) 124, no. 33, 7128–7137, 10.1021/acs.jpcb.0c04553, 32559081. 32559081 PMC7341691

[25] Welch W. , Ruppert J. , and Jain A. N. , Hammerhead: Fast, Fully Automated Docking of Flexible Ligands to Protein Binding Sites, Chemistry & Biology. (1996) 3, no. 6, 449–462, 10.1016/s1074-5521(96)90093-9, 8807875. 8807875

[26] Ekor M. , The Growing Use of Herbal Medicines: Issues Relating to Adverse Reactions and Challenges in Monitoring Safety, Frontiers in Pharmacology. (2014) 4, 10.3389/fphar.2013.00177, 24454289. PMC3887317 24454289

[27] Moise G. , Jîjie A. R. , Moacă E. A. , Predescu I. A. , Dehelean C. A. , Hegheș A. , Vlad D. C. , Popescu R. , and Vlad C. S. , Plants′ Impact on the Human Brain—Exploring the Neuroprotective and Neurotoxic Potential of Plants, Pharmaceuticals. (2024) 17, no. 10, 10.3390/ph17101339. PMC11510325 39458980

[28] Riaz M. , Khalid R. , Afzal M. , Anjum F. , Fatima H. , Zia S. , Rasool G. , Egbuna C. , Mtewa A. G. , Uche C. Z. , and Aslam M. A. , Phytobioactive Compounds as Therapeutic Agents for Human Diseases: A Review, Food Science & Nutrition. (2023) 11, no. 6, 2500–2529, 10.1002/fsn3.3308, 37324906. 37324906 PMC10261751

[29] Knight P. , Chellian R. , Wilson R. , Behnood-Rod A. , Panunzio S. , and Bruijnzeel A. , Sex Differences in the Elevated Plus-Maze Test and Large Open Field Test in Adult Wistar Rats, Pharmacology, Biochemistry, and Behavior. (2021) 204, 173168, 10.1016/j.pbb.2021.173168, 33684454. 33684454 PMC8130853

[30] d′Isa R. , Comi G. , and Leocani L. , The 4-Hole-Board Test for Assessment of Long-Term Spatial Memory in Mice, Current Protocols. (2021) 1, no. 8, 10.1002/cpz1.228. 34432376

[31] Campos-Cardoso R. , Godoy L. D. , Lazarini-Lopes W. , Novaes L. S. , dos Santos N. B. , Perfetti J. G. , Garcia-Cairasco N. , Munhoz C. D. , and Padovan C. M. , Exploring the Light/Dark Box Test: Protocols and Implications for Neuroscience Research, Journal of Neuroscience Methods. (2023) 384, 109748, 10.1016/j.jneumeth.2022.109748, 36410541. 36410541

[32] Pádua-Reis M. , Nôga D. A. , Tort A. B. , and Blunder M. , Diazepam Causes Sedative Rather Than Anxiolytic Effects in C57BL/6J Mice, Scientific Reports. (2021) 11, 10.1038/s41598-021-88599-5. PMC8085115 33927265

[33] Oishi Y. , Saito Y. C. , and Sakurai T. , GABAergic Modulation of Sleep-Wake States, Pharmacology & Therapeutics. (2023) 249, 108505, 10.1016/j.pharmthera.2023.108505, 37541595. 37541595

[34] Bappi M. H. , Prottay A. A. S. , Kamli H. , Sonia F. A. , Mia M. N. , Akbor M. S. , Hossen M. M. , Awadallah S. , Mubarak M. S. , and Islam M. T. , Quercetin Antagonizes the Sedative Effects of Linalool, Possibly Through the GABAergic Interaction Pathway, Molecules. (2023) 28, no. 14, 10.3390/molecules28145616, 37513487. PMC10384931 37513487

[35] Rosas-Sánchez G. U. , German-Ponciano L. J. , and Rodríguez-Landa J. F. , Considerations of Pool Dimensions in the Forced Swim Test in Predicting the Potential Antidepressant Activity of Drugs, Frontiers in Behavioral Neuroscience. (2022) 15, 757348, 10.3389/fnbeh.2021.757348. 35069137 PMC8777187

[36] Guzel T. and Mirowska-Guzel D. , The Role of Serotonin Neurotransmission in Gastrointestinal Tract and Pharmacotherapy, Molecules. (2022) 27, no. 5, 10.3390/molecules27051680, 35268781. PMC8911970 35268781

[37] Page C. E. and Coutellier L. , Kv3.1 Voltage-Gated Potassium Channels Modulate Anxiety-Like Behaviors in Female Mice, Neuroscience. (2024) 538, 68–79, 10.1016/j.neuroscience.2023.12.011, 38157976. 38157976 PMC10872248

[38] Juvenal G. , Higa G. S. V. , Bonfim Marques L. , Tessari Zampieri T. , Costa Viana F. J. , Britto L. R. , Tang Y. , Illes P. , di Virgilio F. , Ulrich H. , and de Pasquale R. , Regulation of GABAergic Neurotransmission by Purinergic Receptors in Brain Physiology and Disease, Purinergic Signal. (2025) 21, no. 1, 149–177, 10.1007/s11302-024-10034-x. 39046648 PMC11958915

[39] Crocetti L. and Guerrini G. , GABAA Receptor Subtype Modulators in Medicinal Chemistry: An Updated Patent Review (2014-Present), Expert Opinion on Therapeutic Patents. (2020) 30, no. 6, 441–454, 10.1080/13543776.2020.1746764. 32200689

[40] Gianni G. and Pasqualetti M. , Wiring and Volume Transmission: An Overview of the Dual Modality for Serotonin Neurotransmission, ACS Chemical Neuroscience. (2023) 14, no. 23, 4093–4104, 10.1021/acschemneuro.3c00648, 37966717. 37966717 PMC10704479

